# Beyond the Bench: COEPs Contribute to Hurricane Relief

**DOI:** 10.1289/ehp.114-a30

**Published:** 2006-01

**Authors:** Tanya Tillett

The conditions in Louisiana and Mississippi following Hurricanes Katrina and Rita reminded us all of our commonality in the human experience and moved many to help. Among those moved to help were the staff at the Community Outreach and Education Programs (COEPs) of NIEHS Centers across the country. Responding to communities in need is one of the primary functions of the COEPs, so providing outreach to those areas on the Gulf Coast impacted by the hurricanes seemed a natural step to take.

“When our director volunteered our COEP [to lead efforts], we remarked that if COEPs had never existed, they would have had to be invented on August 28,” says Pamela Diamond, director of the NIEHS Center COEP at University of Texas Medical Branch (UTMB) in Galveston. Adds Robin Fuchs-Young, director of the COEP of the Center for Research on Environmental Disease at the University of Texas, M.D. Anderson Cancer Center (UTMDACC), “All of us saw what was happening on television and felt compelled on a human level to help in whatever way we could.”

## A Helping Hand

Says Diamond, “Most of the community outreach directors and staff across the country knew one another and trusted one another, and we could quickly organize a response. It was quite a pickup operation—cell phone calls, e-mails in the middle of the night. During our own evacuation due to Hurricane Rita, we sat on [Fuchs-Young’s] back porch, planning supply deliveries, editing public service announcements [PSAs], and identifying scientists in distant states to provide reliable information and data for flyers.”

Two teams from the UTMB COEP were dispatched in early October with different objectives. One team, led by Diamond, connected with shelters in rural LaFourche Parish and delivered humanitarian supplies including first aid equipment, diapers, and drinking water. The other team covered a wider range including Calcasieu, Jefferson, Orleans, Terrebonne, and LaFourche Parishes, as well as Baton Rouge and New Iberia and Port Arthur, Texas, to contact community-based environmental organizations whose operations had been disrupted by the hurricanes. These groups were asked how the events had disrupted their normal functions, what environmental damage they observed, what they saw as the greatest environmental threats facing residents on reentering impacted areas, and how they could unite their skills and networks with scientific and clinical expertise. These interviews were compiled in a DVD format and are being sent to the directors of each COEP and interested personnel at the national level.

The UTMB COEP is also collaborating with the Louisiana Environmental Action Network in funding the preparation and delivery of re-entry hazard protection kits for residents involved in recovery operations. These kits focus on mold and toxic residue hazards and—along with information prepared by the NIEHS, the Centers for Disease Control and Prevention, and the Federal Emergency Management Agency—aim to mitigate citizen exposures.

## Education for the Re-entry Process

The COEPs also recognize that the devastated areas will need resources to help them deal with the long-term environmental aftermath of the hurricanes. Soon after Katrina hit, reports indicated high levels of arsenic and lead in the floodwaters and severe mold contamination. The programs joined forces to provide long-term outreach, and divided into areas of strongest expertise to develop fact sheets offering clear, useful information for citizens in the affected areas.

“The strong desire to return families to their homes and to rebuild neighborhoods needs to be balanced with care to do things right,” says Ruth Woods, program administrator of the Center for Child Environmental Health Risks Research and the Pacific Northwest Center for Human Health and Ocean Studies, both at the University of Washington (UW). “Environmental cleanup needs to be a high priority so that people are not made ill from [environmental exposures].”

The COEPs from UW, the Kresge Center for Environmental Health at Harvard University (in conjunction with Columbia University), the University of Iowa Environmental Health Sciences Research Center (EHSRC), and the Wayne State University Environmental Health Sciences Center in Molecular and Cellular Toxicology with Human Applications have developed fact sheets addressing various elements of returning home safely. Topics include lead and arsenic contamination from floodwaters, mold hazards, and safe cleanup procedures.

Some of the fact sheet material is based on Katrina-specific studies. Peter Thorne, director of the University of Iowa COEP, says members of his group have collected air and surface samples from water-damaged homes in New Orleans. One study showed that the mean airborne endotoxin concentration was 200-fold higher than in nonflooded homes, and levels of airborne mold spores were so high that N95 respirators—devices with a filter efficiency of 95%–are inadequate protection. Thorne says the fact sheets his working group created describe mold hazards and instruct residents on precautions necessary for safe re-entry and cleanup.

To date, the COEPs have distributed more than 67,000 flyers to local leaders in the storm-damaged area. “We are hoping that other . . . COEPs have information on the same or other topics that can be developed into flyers,” says Lisa Pietrantoni, project coordinator for the Wayne State COEP.

What was particularly gratifying about the flyer effort was how clearly the flyers were needed. “I often encountered someone in a shelter who told me they had mold re-entry flyers,” says Diamond. “When we looked at them, they were the flyers that had been created at UW or Wayne State, . . . copied by shelter workers, and passed down the line.”

The COEPs are also using PSAs to get safety information out to residents. The program at the University of New Mexico Center for Environmental Health Sciences produced six PSAs on topics such as safe cleanup methods, water safety, and toxics, and is working with American Forum, a nonprofit media company, to disseminate them to over 3,000 radio, television, and print media outlets in the Gulf Coast area. The UTMDACC COEP is developing PSAs for especially susceptible groups of people, including immunocompromised patients. Still more PSAs may be developed to target specific regional issues and incorporate data that emerge from environmental health studies being conducted. Spanish-language PSAs might also target workers doing the repairs and rebuilding.

## More to Be Accomplished

At the NIEHS Core Centers Annual Meeting held this fall at the Vanderbilt University Center in Molecular Toxicology, COEP staff discussed their outreach efforts and looked ahead to some next steps, such as community forums, town hall sessions, and continued data collection. They concluded that there is still much environmental health aid these towns and cities will need.

One potential partnership that could help the COEPs offer some long-term solutions is the Katrina Environmental Research and Restoration Network (KERRN), a vision conceived by John McLachlan, director of the Center for Bioenvironmental Research at Tulane and Xavier Universities in New Orleans. According to McLachlan, KERRN is “a network of researchers sharing data and ideas, crossing disciplinary, geographical, and institutional boundaries, providing models to respond to and recover from major environmental disasters.” The network, funded by a grant from the National Science Foundation, could be a great help for the residents in the affected area. As Fuchs-Young notes, “Folks in the Gulf Coast want science and data. They want to know what’s going to happen to their water supply and wetlands, and what will be the effect of flooded toxic waste dumps on their lives and livelihoods.”

The communities located throughout the Gulf Coast have a long road ahead of them. There is no question in the minds of most that they can and should rebuild; many have lived in this area for generations, and don’t want to change their way of life. But environmental health experts caution that much care must be taken because of the health threat that contaminants like mold can pose. States Thorne, “There remains extensive remediation work [in the Gulf Coast area] that will expose residents and contractors to mold hazards. The potential for allergy, asthma, and lung infections is high due to the enormous concentrations encountered. It is critically important that residents of Louisiana and Mississippi are protected from these exposures.”

## Figures and Tables

**Figure:**
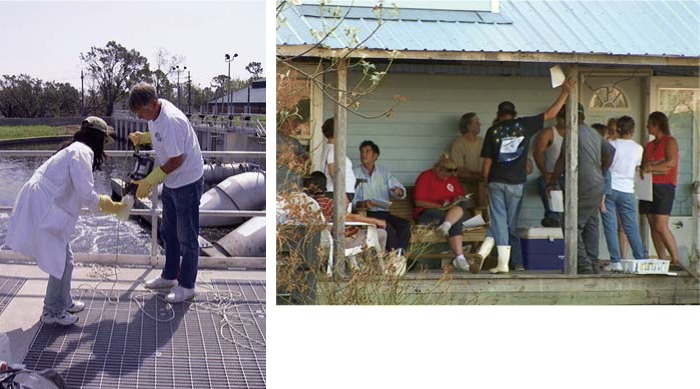
Pitching in. Center staff stepped in at several points, including taking water samples (left, at the 17th Street Canal) and helping area victims sign up for assistance and humanitarian aid (above, at the LaRose community shelter).

